# GWASpro: a high-performance genome-wide association analysis server

**DOI:** 10.1093/bioinformatics/bty989

**Published:** 2018-12-03

**Authors:** Bongsong Kim, Xinbin Dai, Wenchao Zhang, Zhaohong Zhuang, Darlene L Sanchez, Thomas Lübberstedt, Yun Kang, Michael K Udvardi, William D Beavis, Shizhong Xu, Patrick X Zhao

**Affiliations:** 1 Noble Research Institute, Ardmore, OK, USA; 2 AgriLife Research Center, Texas A&M University, Beaumont, TX, USA; 3 Department of Agronomy, Iowa State University, Ames, IA, USA; 4 Department of Botany and Plant Sciences, University of California, Riverside, CA, USA

## Abstract

**Summary:**

We present GWASpro, a high-performance web server for the analyses of large-scale genome-wide association studies (GWAS). GWASpro was developed to provide data analyses for large-scale molecular genetic data, coupled with complex replicated experimental designs such as found in plant science investigations and to overcome the steep learning curves of existing GWAS software tools. GWASpro supports building complex design matrices, by which complex experimental designs that may include replications, treatments, locations and times, can be accounted for in the linear mixed model. GWASpro is optimized to handle GWAS data that may consist of up to 10 million markers and 10 000 samples from replicable lines or hybrids. GWASpro provides an interface that significantly reduces the learning curve for new GWAS investigators.

**Availability and implementation:**

GWASpro is freely available at https://bioinfo.noble.org/GWASPRO.

**Supplementary information:**

Supplementary data are available at *Bioinformatics* online.

## 1 Introduction

Genome-wide association studies (GWAS) for crop improvements often confront significant challenges related to complex experimental designs and large datasets; there is a need for new GWAS analysis software that can address replicated phenotypic data related to complex experimental designs involving multiple environments along with a large-scale molecular marker data. Popular GWAS software tools (Bradbury *et al.*, 2007; Lipka *et al.*, 2012) are confined to a single population and using linear mixed models (LMMs), in particular the QK model, which incorporates both a population stratification structure (Q) matrix and a kinship (K) matrix (Yu *et al.*, 2006). Recently, several modified models, such as the compressed mixed linear model (Zhang *et al.*, 2010), multi-locus mixed model (Segura *et al.*, 2012), FarmCPU (Liu *et al.*, 2016) and the integration of Kruskal–Wallis test with empirical Bayes (pkWemEB) (Ren *et al.*, 2018), were proposed to achieve fast computation and high statistical power. However, all of the above models or software tools lack the capacity to account for the phenotypic variance across environments (Korte and Farlow, 2013). To solve this problem, we present GWASpro, a web-based platform that provides online GWAS data analysis services. GWASpro supports building complex design matrices to account for replicated phenotypic observations (years, treatments, locations and/or replications), which advances the QK model toward better quantitative trait loci (QTL) mapping resolutions. GWASpro is capable of handling a large-scale dataset consisting of up to 10 million markers and 10 000 samples representing the replicable genotypes.

## 2 Methods and implementation

### 2.1 Design matrices

**Fig. 1.**
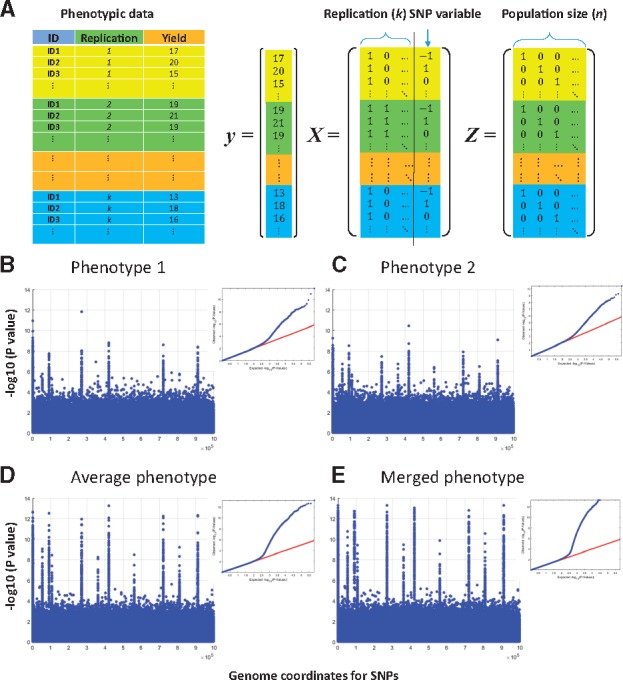
(**A**) Example data and related design matrices for *y*, *X* and *Z*, where *y* is the vector for phenotype, *X* is the design matrix for the fixed effect and *Z* is the design matrix for the random genetic effect. [See Equations (1) and (2) in Supplementary Material A]. (**B**) Manhattan plots and QQ plots, obtained using phenotype 1. (**C**) Manhattan plots and QQ plots, obtained using phenotype 2. (**D**) Manhattan plots and QQ plots, obtained using the average phenotype. (D) Manhattan plots and QQ plots, obtained using the merged phenotype

GWASpro supports flexible building design matrices for the LMM. Figure 1A shows how the design matrices for genotypic data consisting of *m* markers and *n* individuals with *k* replications are arranged.

### 2.2 Efficient computing for large-scale GWAS

In GWASpro, working procedures include building a kinship matrix, fitting the LMM and performing Wald test for calculating *P*-values (Supplementary Material A). GWASpro implements a distributed parallel-computing engine that can effectively utilize ∼1000 CPU cores and ∼10 TB RAM (Supplementary Fig. S1). We also implemented a multi-threading and resumable data-uploading module, utilizing HTML5 protocol for robust and fast data transfer.

### 2.3 Genomic control for adjusting inflated *P*-values

We observed genomic (*P*-value) inflations given a replication factor in our simulation study (Section 3.1) and the Case Study 3. To address this, GWASpro introduces a genomic correction function, by which the inflated *P*-values are adjusted using the genomic inflation factor ($\lambda_{GC}$) as demonstrated by (Devlin and Roeder, 1999; Devlin *et al.*, 2001; van Iterson *et al.*, 2017; Voorman *et al.*, 2011).

### 2.4 Input

GWASpro automatically establishes the LMM with required inputs including a genotypic file, a phenotypic file and variable names with properties (categorical/numerical). Users are responsible for imputations of markers. The upload of kinship matrix is optional as it can be calculated using the genotypic matrix. Missing phenotypic records are automatically excluded. Users can either directly upload data files from a local computer or specify the URLs of user input data, including data sharing URLs of Google Drive and Dropbox for remote downloading using http/https/ftp protocols.

### 2.5 Output

The job queue management system in GWASpro assigns each submission a unique session ID, which can be used to track the job progress and download final results. The GWASpro returns original *P*-values, adjusted *P*-values based on genomic control, QQ plot and Manhattan plot.

## 3 Results and discussion

### 3.1 Simulation study: assessing QTL mapping resolution

Our simulated dataset mimics a situation in which two identical plant populations (A and B) are grown in two environments (Supplementary Material C). We prepared four phenotypic datasets: phenotype 1 (Fig. 1B), phenotype 2 (Fig. 1C), average phenotype (Fig. 1D) and merged phenotype (Fig. 1E). Heritability for each population was adjusted to 0.5. The principle of this simulation was introduced in (Kim, 2017). The resulting Manhattan plots reveal that Figure 1E produces the best QTL resolution with the highest QTL peaks and trivial background inflation, followed by Figure 1D. To compare the analysis performance between Figure 1D and E, the receiver operating characteristic curves were drawn (Supplementary Fig. S5). The area under the curves for Figure 1D and E were 0.9178 and 0.9276, respectively. This supports that Figure 1E shows better QTL resolution. This is a novel benefit of GWASpro, suggesting that accounting for the phenotypic variations can improve QTL mapping resolution by reducing the missing heritability (Korte and Farlow, 2013).

### 3.2 Case study 1: comparing GWASpro, GAPIT and PEPIS

We analyzed the thousand-grain weight (as phenotype) for the IMF2 rice population (Hua *et al.*, 2002, 2003) using GAPIT (Lipka *et al.*, 2012), PEPIS (Zhang *et al.*, 2016) and GWASpro (Supplementary Fig. S2). All significant peaks were consistent. In particular, GAPIT and GWASpro yielded similar plot outlines with different *P*-value scales, which indicates that different *P-*value thresholds must be applied to the GAPIT and GWASpro results. GAPIT, PEPIS and GWASpro have different characteristics: GAPIT should be used for a single population in the additive QK model; PEPIS for a single population accounting for additive, epistasis and dominant effects in the K model and GWASpro for either a single or replicated genotypes in either the K or QK model.

### 3.3 Case study 2: *Medicago truncatula* data

Kang *et al.* (2015) published GWAS results for leaf size and shoot biomass weight traits with a *Medicago truncatula* HapMap population consisting of 220 accessions with 1 810 466 SNPs using TASSEL (Supplementary Material B). We re-analyzed the same dataset using GWASpro and TASSEL and compared their results. The resulting Manhattan plots and QQ plots are very similar to each other (Supplementary Fig. S3).

### 3.4 Case study 3: maize data

Sanchez *et al.* (2018) published GWAS results with three replicated populations (302 maize accessions in each population) using GAPIT. We analyzed the same data using GWASpro. GAPIT and GWASpro produced different results because the GWASpro results were obtained directly using the replicated phenotypic data, whereas the GAPIT results were obtained using the breeding values (BVs) predicted from the replicated genotypes. The authors used the BVs for GWAS analyses because GAPIT is not capable of handling the replications. GAPIT required twice fitting the LMMs for BV prediction and GWAS, which might cause LMM overfitting. With GWASpro, this problem can be avoided. The genomic inflation was observed in the GWASpro results, which is common given replicated genotypes (Ehret, 2010; van Iterson *et al.*, 2017; Voorman *et al.*, 2011). To address this issue, the population stratification resulting from the principle component analysis was first accounted for then, *P*-vaules were adjusted by the genomic control (Section 2.3) in our analysis. Supplementary Figure S4 compares the results obtained by GWASpro and GAPIT.

### 3.5 Performance test

We performed benchmark tests of GWASpro by measuring runtimes (Supplementary Table S1) given the various sizes of data (1 million, 3 million, 5 million, 10 million SNPs; 1k, 3k, 5k individuals). Supplementary Figure S6 summarizes that the runtime generally increases following $O(n^{2}m)$, where *n* is sample size and *m* is marker size.

## 4 Conclusion

GWASpro is an online platform for GWAS analysis that does not require the hassles of software installation and maintenance. The parallel computing engine allows GWASpro to quickly analyze a large-scale dataset. In GWASpro, the QK model is implemented for unbiased QTL mapping by accounting for the kinship matrix (K) and population stratification (Q) (Yu *et al.*, 2006). GWASpro can address replicated phenotypic data, which are typically from self-pollinating plant species. Our simulation datasets demonstrate that GWASpro captures the amplified QTL signals when the gene-environment interactions in multiple replications are in similar patterns. Our Maize datasets demonstrate that GWASpro captures QTLs by accounting for the phenotypic variations across different environments. The environmental factors are crucial to identify robust environment-resistant QTL (Palomeque *et al.*, 2010; Xavier *et al.*, 2018). In addition, GWASpro supports BV estimation, which is introduced in Supplementary Material D.

## Funding

This work was supported by the National Science Foundation collaborative research grant award DBI-1458597 to P.X.Z. and DBI-1458515 to S.X.; and by partial funding support from the Noble Research Institute to P.X.Z.; the North Central Soybean Research Program, Baker Center for Plant Breeding, USDA-NIFA project IOW04314 and the GF Sprague Endowment of the Agronomy Department at Iowa State University to W.D.B.

*Conflict of Interest*: none declared.

## Supplementary Material

bty989_Supplementary_Materials
