# Supplementary material for: GWASpro: a high-performance genome-wide association analysis server
Source: Bioinformatics. 2018 Dec 3;35(14):2512–4. doi: 10.1093/bioinformatics/bty989 (PMC6612817; doi:10.1093/bioinformatics/bty989)
Supplement: bty989_Supplementary_Materials [file bty989_supplementary_materials.docx]

**Supplementary Materials (A-D)**

GWASpro: A High-Performance Genome-Wide Association Analysis Server

Bongsong Kim^1^, Xinbin Dai^1^, Wenchao Zhang^1^, Zhaohong Zhuang^1^, Darlene L. Sanchez^2^, Thomas Lübberstedt^3^, Yun Kang^1^, Michael Udvardi^1^, William D. Beavis^3^, Shizhong Xu^4*^ and Patrick X. Zhao^1*^

^1^ Noble Research Institute, Ardmore, OK 73401, USA

^2^ Texas A&M AgriLife Research, Beaumont, TX 77713, USA

^3^ Department of Agronomy, Iowa State University, Ames, IA 50011, USA

^4^ Department of Botany and Plant Sciences, University of California, Riverside, CA 92521, USA

^*^To whom correspondence should be addressed.

*Corresponding Authors:

Shizhong Xu, [shizhong.xu@ucr.edu](mailto:shizhong.xu@ucr.edu); phone: +1-951-827-5898;

Patrick X. Zhao, [pzhao@noble.org](mailto:pzhao@noble.org); Phone: +1-580-224-6725

Running Title: GWASpro: Genome-Wide Association Analysis Server

# Supplementary Material A: Statistical backgrounds

**Linear mixed model**

The linear mixed model (LMM) includes both fixed and random genetic effects and can be formulated as:

*y = X$\beta$ + Zu + ε* (1)

where *y* is the vector for phenotype; *X* is the design matrix for the fixed effect; *Z* is the design matrix for the random genetic effect; $\beta$ is the vector for the fixed effect; *u* is the vector for the random genetic effect; and *ε* is the vector for residuals.

Individuals in a population are assumed to have a genetic variance-covariance structure, which can be expressed as a genetic relationship matrix, *K*. The LMM can be denoted as:

$\left( \begin{matrix} X^{'}X & X^{'}Z \\ Z^{'}X & Z^{'}Z+{\frac{\sigma_{\varepsilon}^{2}}{\sigma_{u}^{2}}K}^{-1} \end{matrix} \right)\left( \begin{matrix} \hat{\beta} \\ \hat{u} \end{matrix} \right)$ *=* $\left( \begin{matrix} X^{'}y \\ Z^{'}y \end{matrix} \right)$ (2)

where $\sigma_{u}^{2}$ is the variance component for *u*; $\sigma_{\varepsilon}^{2}$ is the variance component for *ε*; $\hat{\beta}$ is the estimated fixed effect; $\hat{u}$ is the predicted random genetic effect; *y* is a vector of phenotypic values. In Equation 2, the random genetic effect is assumed that *u* ~ *N*(0, $K\sigma_{u}^{2}$). The residual errors are assumed that *ε* ~ *N*(0, $I\sigma_{\varepsilon}^{2}$). In order to calculate $\hat{\beta}$ and $\hat{u}$, the values for $\sigma_{\varepsilon}^{2}$ and $\sigma_{u}^{2}$ must be approximated. GWASpro implements two kinds of restricted maximum likelihood (REML) algorithms: the expectation-maximization (EM) algorithm and the Newton iterative optimization algorithm.

**The EM algorithm for breeding value estimation**

We implemented the EM algorithm for estimating breeding values (BVs).

Let us rewrite Equation 2 as:

$\left( \begin{matrix} C_{11} & C_{12} \\ C_{21} & C_{22}+{\frac{\sigma_{\varepsilon}^{2}}{\sigma_{u}^{2}}K}^{-1} \end{matrix} \right)\left( \begin{matrix} \hat{\beta} \\ \hat{u} \end{matrix} \right)$ *=* $\left( \begin{matrix} W_{1} \\ W_{2} \end{matrix} \right)$ (3)

For the EM algorithm, GWASpro implements the row operation. $\hat{\beta}$ and $\hat{u}$ can be calculated as:

$\hat{\beta}$ *=* ${(C_{11}-C_{12}\left( C_{22}+{\frac{\sigma_{\varepsilon}^{2}}{\sigma_{u}^{2}}K}^{-1} \right)^{-1}C_{21})}^{-1}$ ($W_{1}-C_{12}\left( C_{22}+{\frac{\sigma_{\varepsilon}^{2}}{\sigma_{u}^{2}}K}^{-1} \right)^{-1}W_{2}$) (4)

$\hat{u}$*=* $({C_{22}+{\frac{\sigma_{\varepsilon}^{2}}{\sigma_{u}^{2}}K}^{-1})}^{-1}$($W_{2}-C_{21}\hat{\beta}$) (5)

In Equations 4 and 5, $\left( C_{22}+{\frac{\sigma_{\varepsilon}^{2}}{\sigma_{u}^{2}}K}^{-1} \right)^{-1}$ can be rewritten as:

$\left( I+\frac{\sigma_{\varepsilon}^{2}}{\sigma_{u}^{2}}{(KC_{22})}^{-1} \right)^{-1}{C_{22}}^{-1}$ (6)

If $KC_{22}$ is not singular, $KC_{22}$ = $UDU'$ based on the eigenvalue decomposition so that Equation 6 can be written as:

${U\left( I+\frac{\sigma_{\varepsilon}^{2}}{\sigma_{u}^{2}}D \right)}^{-1}U'{C_{22}}^{-1}$ (7)

Therefore, Equation 4 can be rewritten as:

$\hat{\beta}$ *=* ${(C_{11}-C_{12}U\left( I+\frac{\sigma_{\varepsilon}^{2}}{\sigma_{u}^{2}}D \right)^{-1}U'{{C_{22}}^{-1}C}_{21})}^{-1}$ ($W_{1}-C_{12}U\left( I+\frac{\sigma_{\varepsilon}^{2}}{\sigma_{u}^{2}}D \right)^{-1}U'{{C_{22}}^{-1}W}_{2}$) (8)

As $\frac{\sigma_{\varepsilon}^{2}}{\sigma_{u}^{2}}$ is updated at each iteration, the convergences for $\hat{\beta}$ and $\hat{u}$ are reached. For faster computation, Equations 4 and 5 can be efficiently calculated by: (1) precalculating the repeatedly used matrices during the iterations; and (2) using $\frac{\sigma_{u}^{2}}{\sigma_{u}^{2}+ \sigma_{\varepsilon}^{2}D_{(i,i)}}$ for calculating $\left( I+\frac{\sigma_{u}^{2}}{\sigma_{\varepsilon}^{2}}D \right)^{-1}$, where $D_{(i,i)}$ is the *i^th^* diagonal element of $D$. Equation 4 can be rewritten as:

$\hat{\beta}$ *=* ${(C_{11}-M_{1}HM_{2})}^{-1}$ ($W_{1}-M_{1}HM_{3}$) (9)

where $M_{1}$ = $C_{12}U$;$H$ = a diagonal matrix in which $H_{(i,i)}=\frac{\sigma_{u}^{2}}{\sigma_{u}^{2}+ \sigma_{\varepsilon}^{2}D_{(i,i)}}$; $M_{2}$ = $U'{{C_{22}}^{-1}C}_{21}$;$M_{3}$ = $U'{C_{22}}^{-1}W_{2}$.

If $C_{22}$ is an identity matrix, Equation 9 will be equivalent to:

$\hat{\beta}$ *=* ${(C_{11}-M_{1}HM_{1}^{'})}^{-1}$ ($W_{1}-M_{1}HM_{3}$) (10)

Equation 5 can be rewritten as:

$\hat{u}$*=* $UHM_{4}$ ($W_{2}-C_{21}\hat{\beta}$) (11)

where $M_{4}$ = $U'{C_{22}}^{-1}$.

The two variances, $\sigma_{\varepsilon}^{2}$ and $\sigma_{u}^{2}$, can be calculated using the following equations:

$\sigma_{\varepsilon}^{2}=\frac{y^{'}(y-X\beta-Zu)}{n-q}$ (12)

$\sigma_{u}^{2}=\frac{\hat{u}^{'}K^{-1}\hat{u}+\sigma_{\varepsilon}^{2}\mathrm{tr}{(\frac{\sigma_{\varepsilon}^{2}}{\sigma_{u}^{2}}I+{K(C}_{22}-C_{21}{C_{11}}^{-1}C_{12}))}^{-1}}{q}$ (13)

where *n* = the number of rows of *X* and *q* = the number of columns of *X*; other terms are described above.

Based on the row operation, a matrix in a size of $n^{2}$ can be split into ${n_{1}}^{2}$ and ${n_{2}}^{2}$, in which $n_{1}$ is the number of columns in the *X* matrix and $n_{2}$ is the number of the columns in the *Z* matrix (see Equation 1). Therefore, the computing burden can be substantially reduced. Based on the eigenvalue decomposition, a procedure to invert a matrix can be simplified, which accelerates computing speed. Given relatively large data sets (e.g. population size > 10,000), the EM algorithm uses less memory and is faster than the Newton iterative algorithm.

**The Newton iterative algorithm for GWAS**

We implemented the Newton iterative method for GWAS.

Given λ = $\sigma_{u}^{2}/\sigma_{\varepsilon}^{2}$, the REML solutions for $\hat{\beta}$ and $\hat{u}$ in Equation 2 are:

$\hat{\beta}={(X'V_{0}^{-1}X)}^{-1}X'V_{0}^{-1}y$ (14)

$\hat{\sigma}_{\varepsilon}^{2}$ = $\frac{1}{n-p}{(y-X\hat{\beta})}^{T}V_{0}^{-1}(y-X\hat{\beta})$ (15)

Note that $V_{0}$ is still unknown and can be written as:

$V_{0}^{-1}$ = ${(ZKZ'\lambda+ I)}^{-1}$ (16)

Based on the eigenvalue decomposition, $ZKZ'$ can be expressed as $UDU^{'}$, in which *U* is an orthogonal matrix (eigenvector) and *D* is a diagonal matrix with real positive numbers (eigenvalues). Thus, Equation 16 can be rewritten as:

$V_{0}^{-1}$ = $U{(\lambda D+I)}^{-1}U'$ (17)

where ${(\lambda D+I)}^{-1}$ can be calculated by:

$\frac{1}{(1+\lambda D_{(i,i)})}$ (18)

Parameter $\lambda$ can be solved by maximizing the following restricted likelihood function:

*L*(λ) =$-\frac{1}{2}\ln\left| V_{0} \right|-\frac{1}{2}\left| X^{'}V_{0}^{-1}X \right|-\frac{1}{2}(n-p) \ln\left| \hat{\sigma}_{\varepsilon}^{2} \right|$ (19)

Since this likelihood function only contains one unknown parameter, $\lambda$, we can solve it using the Newton iterative algorithm.

**Wald test and *p-*value estimation**

To test the significance of a marker, we used the Wald test. Recall that one of the fixed effects is the effect of marker *k*. Let us partition the fixed effects into $\beta=\left[\begin{matrix} \beta_{0}^{T} & \beta_{k} \end{matrix}\right]^{T}$ where $\beta_{0}$ is a vector of fixed effects that exclude the effect of marker *k* and $\beta_{k}$ is the effect of the *k*^th^ marker. Let us also partition the design matrix of the fixed effects into $X=\left[\begin{matrix} X_{0} & Z_{k} \end{matrix}\right]$ where $X_{0}$ is the design matrix for all fixed effects except the genotypes for marker *k* and $Z_{k}$ is the genotype indicator variable for marker *k* (a vector). Therefore, we can write the model as:

$y=X\beta$ *+ Zu + ε = $X_{0}\beta_{0}+Z_{k}\beta_{k}$ + Zu + ε* (20)

The estimated fixed effects are:

$\left[\begin{matrix} \hat{\beta}_{0} \\ \hat{\beta}_{k} \end{matrix}\right]=\left[\begin{matrix} X_{0}^{T}V_{0}^{-1}X_{0} & X_{0}^{T}V_{0}^{-1}Z_{k} \\ Z_{k}^{T}V_{0}^{-1}X_{0} & Z_{k}^{T}V_{0}^{-1}Z_{k} \end{matrix}\right]^{-1}\left[\begin{matrix} X_{0}^{T}V_{0}^{-1}y \\ Z_{k}^{T}V_{0}^{-1}y \end{matrix}\right]$ (21)

# The variance matrix of the estimated fixed effects is:

$var\left[ \begin{aligned} \hat{\beta}_{0} \\ \hat{\beta}_{k} \end{aligned} \right]$ = $\left[ \begin{matrix} var(\hat{\beta}_{0}) & cov(\hat{\beta}_{0},\hat{\beta}_{k}) \\ cov(\hat{\beta}_{k},\hat{\beta}_{0}) & var(\hat{\beta}_{k}) \end{matrix} \right]= \left[ \begin{matrix} X_{0}^{T}V_{0}^{-1}X_{0} & X_{0}^{T}V_{0}^{-1}Z_{k} \\ Z_{k}^{T}V_{0}^{-1}X_{0} & Z_{k}^{T}V_{0}^{-1}Z_{k} \end{matrix} \right]$ $\hat{\sigma}_{\varepsilon}^{2}$ (22)

The Wald test for marker *k* is:

$W_{k}=\frac{\hat{\beta}_{k}^{2}}{\mathrm{var}(\hat{\beta}_{k})}$ (23)

# Under the null hypothesis, $H_{0}:\beta_{k}=0$, $W_{k}$ approximately follows a Chi-square distribution with one degree of freedom. Therefore, the *p*-value can be obtained using:

# $p_{k}=1-\Pr(\chi_{1}^{2}<W_{k})$ (24)

# where $\chi_{1}^{2}$ is a Chi-square variable with one degree of freedom.

# Implementation

# GWASpro conducts GWAS analyses and BV estimation using its backend computational modules written in C++, and those modules are streamlined using R, Linux Shell Script, JAVA, and Python. In GWASpro, Armadillo C++ library (Sanderson, 2010) is used for computing linear systems; C++ code for the ‘optim’ function from the R package (R Core Team, 2014) is used to conduct the Newton iterative algorithm; about 1,000 CPU cores and 10 TB RAM are used for parallel computation; repeated phenotypes related to experimental designs can be handled; and the Wald test is used for the hypothetical test. Figure S1 shows the workflow of the GWASpro analysis procedures.

**Functionalities of GWASpro**

GWASpro automatically excludes phenotype rows that include missing values. Users are responsible for imputing missing values in genotypic data. The genetic relationship matrix (*K* matrix) is a crucial component in the LMM. In GWASpro, either externally imported or internally calculated *K* matrix can be used. GWASpro internally supports computing the *K* matrix based on genotypic data. Given a large genotype data, computing the *K* matrix requires much memory. In order to efficiently manage the memory, the forward chopping algorithm (Kim and Beavis, 2017) is introduced to VanRaden’s algorithm (VanRaden, 2008) as shown below:

*K* = $\frac{\sum_{i=1}^{n} X_{i}^{ʹ}X_{i}}{\sum_{i=1}^{n} w_{i}}$ (25)

where *K* is the genetic relationship matrix; *i* is the loop variable; *n* is the total number of pieces of divided genotypes; $X_{i}$ is the *i*^th^ divided piece of genotype; $w_{i}$ is the number of markers in a piece of *i*^th^ divided genotype.

Other software tools are also available to compute the *K* matrix, e.g. TASSEL (Bradbury, et al., 2007), *Numericware N* (Kim, et al., 2016), and *Numericware i* (Kim and Beavis, 2017). In LMM, the fixed-effect variables can be either categorical or numerical. The variables for treatments, replications, years, and locations must be categorical, while multidimensional vectors resulting from the principle component analysis (PCA) must be numerical.


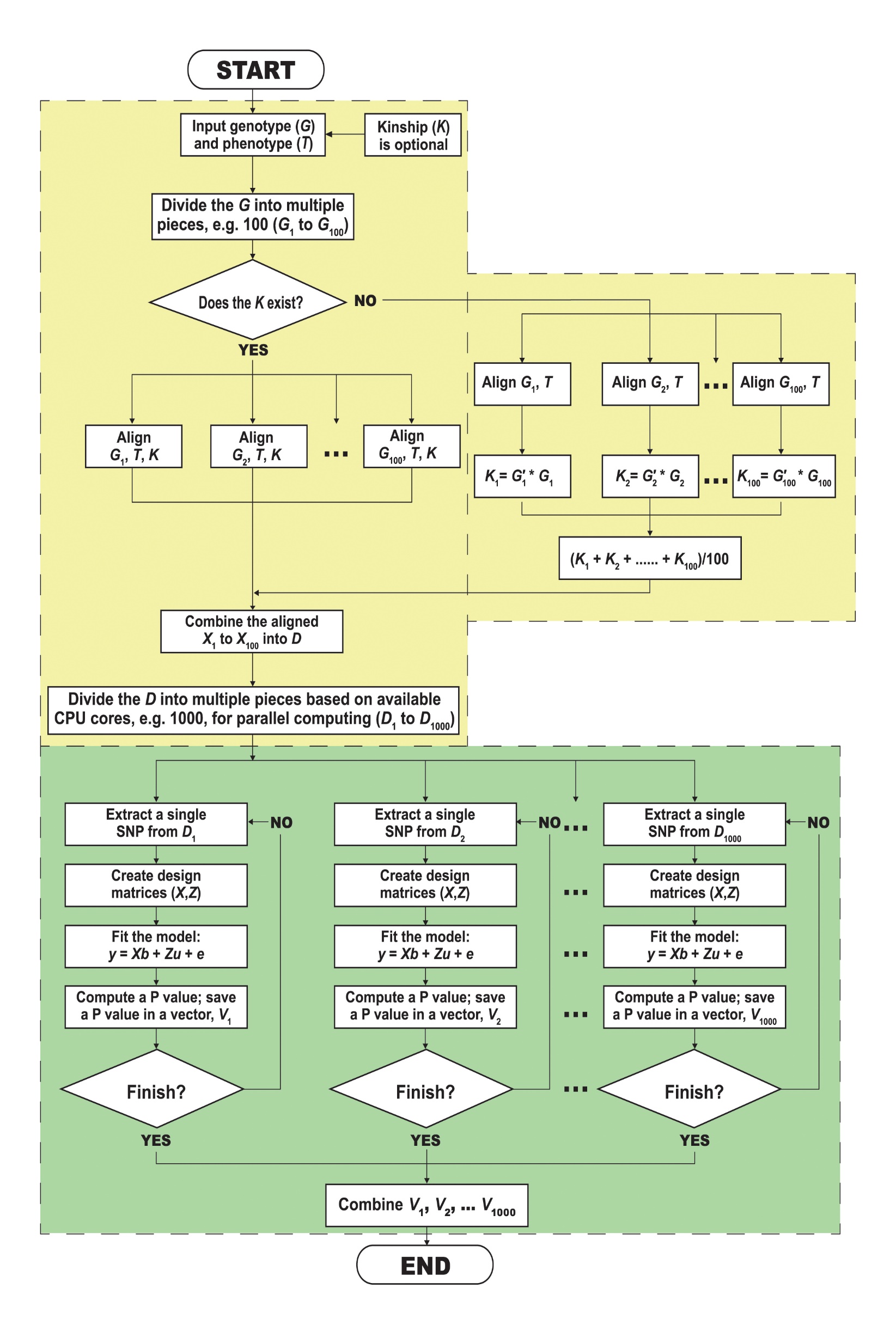


Preprocess

GWAS process

**Figure S1.** The GWASpro workflow illustrates the whole computing process.

**Supplementary Material B: Case studies**

**Case study 1: Results obtained by GWASpro, GAPIT, and PEPIS given the rice data**

The RIL-generated crosses immortal F2 (IMF2) rice population was used for the purpose of testing the reliability of GWASpro in comparison with GAPIT (Lipka, et al., 2012) and PEPIS (Zhang, et al., 2016) (**Figure S2**). The population generation method is introduced in previous papers (Hua, et al., 2003; Hua, et al., 2002). The population size is 278, and the marker set size is 1,619. Each marker has three types of scores (-1, 0, 1), representing categorized types of genomic information on a single chromosomal bin harnessing multiple SNPs. The method for converting a single chromosomal bin into a single marker score is described in previous papers (Xie, et al., 2010; Yu, et al., 2006). The trait for the phenotypic data is thousand-grain weight (KGW). The tests for evaluating the trait were replicated twice over two consecutive years, 1998 and 1999. The two phenotypes for the same populations grown over different years were reduced into a single phenotype by removing the year effects based on the following method introduced in (Xu, 2013):

$y_{j}=\frac{1}{2}[\left( y_{j1}-\bar{y}_{1} \right)+\left( y_{j2}-\bar{y}_{2} \right)]$ (26)

where $\bar{y}_{1}$ and $\bar{y}_{2}$ are the mean phenotypes for 1998 and 1999, respectively.


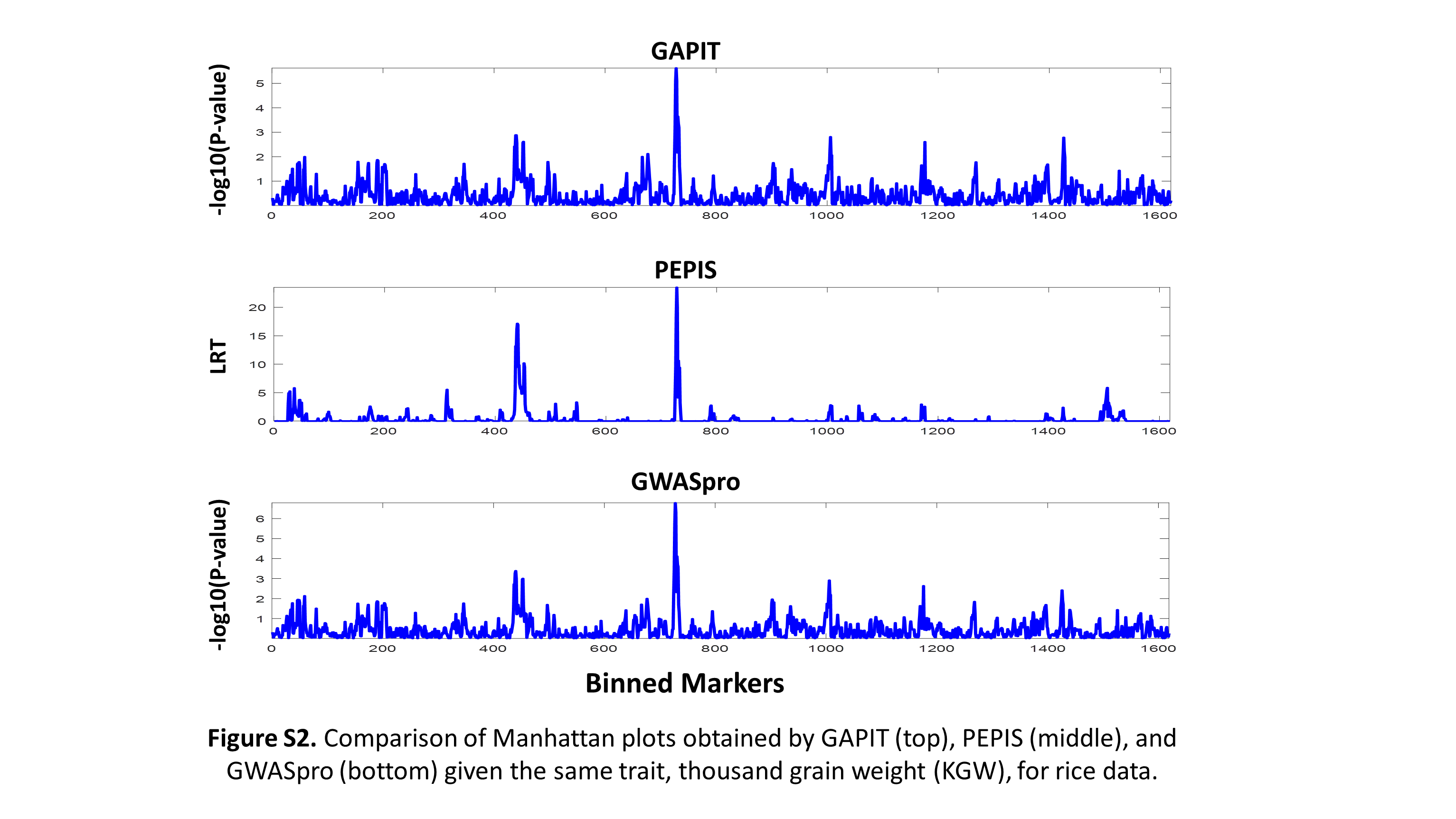


**Figure S2.** Comparison of Manhattan plots obtained by GAPIT (top), PEPIS (middle), and GWASpro (bottom) given the thousand-grain weight (KGW) trait, for the rice data set.

**Case study 2: Results obtained by GWASpro and TASSEL given the *Medicago truncatula* data**

A HapMap for a *Medicago truncatula* population containing 220 accessions was analyzed with two traits: shoot dry weight and leaf size (**Figure S3**). We conducted SNP imputation based on the LD-kNNi algorithm (Money, et al., 2015) using TASSEL. The number of SNPs was 1,810,466. GWAS analyses were performed using TASSEL in the same manners as the original study (Kang, et al., 2015). The HapMap format was converted into the numeric format of 0, 1, and 2 using TASSEL, in which 0 and 2 represent two different homozygote alleles and 1 represents a heterozygote allele. The numeric form of SNPs was then loaded into GWASpro for the same GWAS analyses. The Manhattan plots were generated using the “qqman” library (Turner, 2014) of the R package (Team, 2014). The LMM was formulated as:

$y_{i}$ = μ + $g_{i}$+ $Q_{1}$+ ε

where $y_{ij}$ = the phenotypic value for *i^th^* individual; *μ* = the overall mean; $g_{i}$ = the *i*th random genetic effect; $Q_{1}$ is the first column of STRUCTURE results; and *ε* is the residual.

**
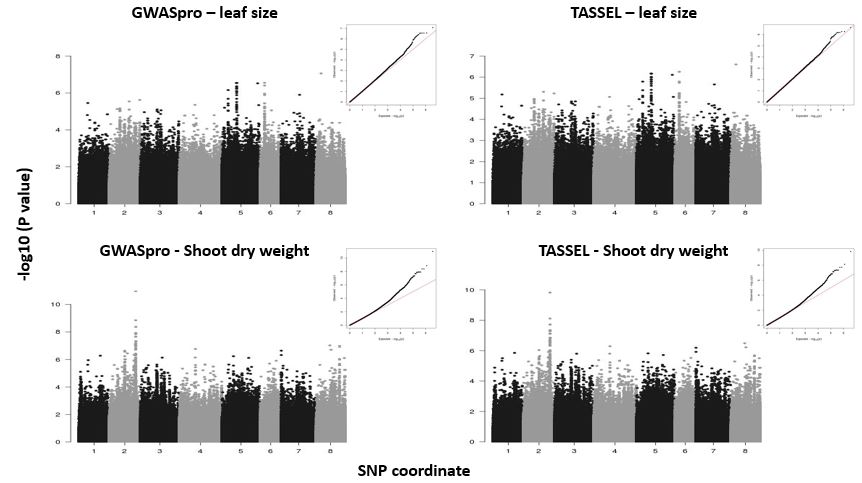
**

**Figure S3.** Manhattan plots and QQ plots obtained by GWASpro and TASSEL for leaf size and shoot dry weight given the *Medicago truncatula* HapMap accessions.

**Case study 3:** **Results obtained by GWASpro and GAPIT given the Maize data set**

Root system architecture (RSA) traits of 302 maize lines (300 doubled haploid lines and two elite inbred lines, PHB47 and PHZ51) from the study of (Sanchez, et al., 2018) were analyzed for GWAS using GWASpro (**Figure S4**). A total of 62,077 biallelic genotyping by sequencing (GBS) markers were used in the analyses. The genotypic data was converted to numerical format using GAPIT (Lipka, et al., 2012). The traits analyzed were total number of roots (TNR) and total root length (TRL). The phenotypic data set includes three replications, and principal component analysis (PCA) was done using GAPIT. The replication factor and the first three columns of the principal components were accounted for as fixed effect terms. Therefore, the LMM was formulated as:

$y_{ij}$ = μ +${rep}_{j}$+ $g_{ij}$+${PCA}_{1,2,3}$+ε

**
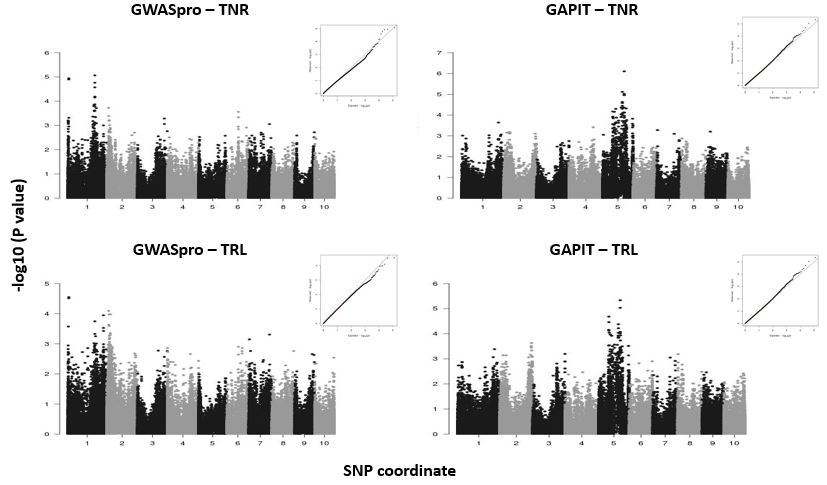
**

**Figure S4.** Manhattan plots and QQ plots obtained by GWASpro and GAPIT for total root length (TRL) and total number of roots (TNR) given the Maize population.

**Supplementary Material C: Simulations for sensitivity evaluations and benchmarks**

**Simulation of a single population**

Simulated data sets were generated based on the foundation of Kim’s simulation method for explaining the hierarchical association coefficient algorithm (Kim, 2017). The simulation procedures are as follows:

1. Create a data set X with *n* (e.g. 1,000) individuals and *m* (e.g. 1,000,000) SNPs consisting of 0, 1, and 2, representing ‘aa,’ ‘Aa,’ and ‘AA’ genotypes, respectively.

2. Set genotypic scores vs. marker-effect values as follows: 0: 1: 2 = 0.02: 0.03: 0.04. This is based on the assumption that ‘A’ and ‘a’ increase phenotypic values by 0.02 and 0.01, respectively.

3. Make triangles on the top edge and triangles on the bottom edge in the data set generated in Step 1. Fill the triangles on the top edge with 0s and triangles on the bottom edge with 2s.

4. Calculate a phenotypic value for each individual by summing up all marker-effect values based on the ratio defined in Step 2. Note that because no errors are assumed in the model, the data set generated from Steps 1 through 4 has heritability (*H^2^*) of 1.0 based on the following equation:

$H^{2}$ = $\frac{\sigma_{u}^{2}}{\sigma_{u}^{2}+ \sigma_{\varepsilon}^{2}}$ (27)

where $H^{2}$ is the broad-sense heritability; $\sigma_{u}^{2}$ is the variance component for random genetic effect; and $\sigma_{\varepsilon}^{2}$ is the variance component for residual effect. To control the value for $\sigma_{\varepsilon}^{2}$, Equation 27 can be rearranged as follows:

$\sigma_{\varepsilon}^{2}= \left( \frac{1}{H^{2}}-1 \right)\sigma_{u}^{2}$ (28)

Note that the $H^{2}$ can range between 0 and 1. By controlling the value for $H^{2}$, the value for $\sigma_{\varepsilon}^{2}$ can be adjusted.

6. To set the Heritability at 0.5, substitute a value of 0.5 for the $H^{2}$ in Equation 28. So the values for $\sigma_{\varepsilon}^{2}$ and $\sigma_{u}^{2}$ become equal. Then randomly generate 1,000 values conforming *N*(0, $\sigma_{\varepsilon}^{2}$) and add the 1,000 random numeric values to the phenotype.

**Simulation of two identical populations**

In order to mimic two phenotypes attached to the same populations tested across two different environments, the R code for the above steps was conducted twice. In the second round, the ratio in Step 2 was differently set as:

-1: 0: 1 = 0.0202: 0.0404: 0.0606

From the second round, we only took the phenotype and dropped the genotype because we needed the two phenotypes attached to the same genotype. We standardized each phenotype corresponding to each environment by calculating z-scores as follows:

$z=\frac{x- \mu}{\sigma}$ (29)

where *z* is the z-score; *x* is the phenotypic value; $\mu$ is the phenotypic mean for a population; and $\sigma$ is the standard deviation derived from phenotypic values within a population.

As introduced in the main manuscript, the QTL mapping resolution was the best at the phenotype that merges the two phenotypes, followed by the average phenotype. To compare the sensitivities between the averaged phenotype and the merged phenotype, the Receiver Operating Characteristic (ROC) curves were drawn, and the areas under each curve were compared (**Figure S5**).


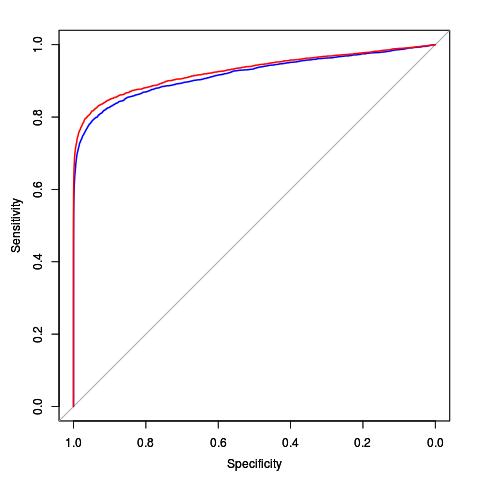


**Figure S5.** ROC curve showing the comparison of sensitivities between the merged phenotype (red) and average phenotype (blue). The areas under curve were 0.9276 and 0.9178, respectively.

**Benchmarks**

We performed benchmark tests using GWASpro with simulated data sets of various sizes (1 million, 3 million, 5 million, 10 million SNPs; 1k, 3k, 5k individuals) and measured runtimes (**Table S1**). **Figure S6** shows that the resulting runtime generally increases following $O(n^{2}m)$, where *n* is sample size and *m* is marker size.

**Table S1.** Computing time given various sizes of SNP sets (1 million, 3 million, 5 million, and 10 million SNPs) and population sizes (1k, 3k, and 5k).

|  | **SNP Markers** | | | | | | | | | |
| --- | --- | --- | --- | --- | --- | --- | --- | --- | --- | --- |
|  | **1,000,000** | | | **3,000,000** | | **5,000,0000** | | | **10,000,000** | |
| **Individuals** | *Pre-process*† | *GWAS process* ‡ | | *Pre-process*  † | *GWAS process* ‡ | *Pre-process* † | | *GWAS process* ‡ | *Pre-process* † | *GWAS process* ‡ |
|  | *Total time (second)* | | | *Total time (second)* | | *Total time (second)* | | | *Total time (second)* | |
| **1,000** | 308 | | 257 | 580 | 413 | 872 | 397 | | 1,708 | 638 |
|  | 565 | | | 894 | | 1,269 | | | 2,346 | |
| **3,000** | 794 | | 3,661 | 2,380 | 4049 | 3381 | 4412 | | 7,196 | 5,668 |
|  | 4,455 | | | 6,429 | | 7,793 | | | 12,864 | |
| **5,000** | 1,786 | | 16,276 | 4,373 | 16,994 | 7,498 | 17,499 | | 12,921 | 20,025 |
|  | 18,062 | | | 21,367 | | 24,997 | | | 32,946 | |

†: The procedure includes aligning multiple input data and computing a kinship matrix (see **Figure S1)**.

‡: The procedure includes fitting the LMM and conducting the Wald test (see **Figure S1)**.

**Figure S6.** Plot showing the runtimes of GWAS analyses given population sizes (1k, 3k, and 5k individuals) and various marker set sizes (1 million, 3 million, 5 million, and 10 million SNPs).

# Supplementary Material D: Estimating breeding values

The GWASpro also supports breeding value estimation. **Figure S7** represents the comparison between estimated breeding values (EBVs) calculated by the Newton iterative algorithm (GWASpro), the EM algorithm (GWASpro), and SAS®, being identical to one another when applied to the same data set. The variance components for the EBVs and residuals from all methods are shown in Table S2, indicating that the results calculated by the EM and Newton iterative algorithms in GWASpro are nearly the same as the result from SAS®. **Table S2** and **Figure S7** indicate that GWASpro produces reliable results.

**Table S2.** Comparisons of variance components for EBVs ($\hat{\sigma}_{u}^{2}$) and for residuals ($\hat{\sigma}_{\varepsilon}^{2}$).

| **Variance components** | **SAS®** | **GWASpro** | |
| --- | --- | --- | --- |
|  |  | **REML** | **EM** |
| $\hat{\sigma}_{u}^{2}$ | 23.711 | 23.712 | 23.712 |
| $\hat{\sigma}_{\varepsilon}^{2}$ | 33.480 | 33.480 | 33.480 |


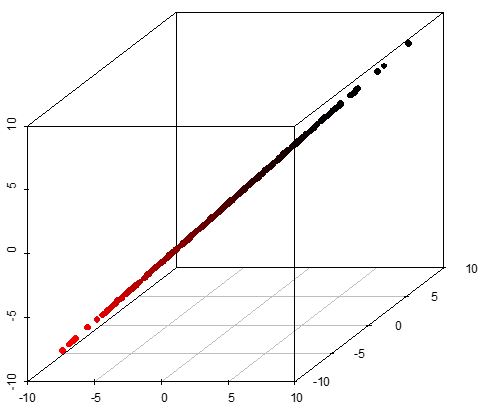


SAS

Expectation-maximization algorithm

*Newton iterative algorithm*

**Figure S7.** Three-dimensional correlation plot of estimated breeding values calculated by SAS®, the expectation-maximization algorithm (GWASpro), and the Newton iterative algorithm (GWASpro).

**References**

Bradbury, P.J.*, et al.* TASSEL: software for association mapping of complex traits in diverse samples. *Bioinformatics* 2007;23(19):2633-2635.

Hua, J.*, et al.* Single-locus heterotic effects and dominance by dominance interactions can adequately explain the genetic basis of heterosis in an elite rice hybrid. *Proceedings of the National Academy of Sciences of the United States of America* 2003;100(5):2574-2579.

Hua, J.P.*, et al.* Genetic dissection of an elite rice hybrid revealed that heterozygotes are not always advantageous for performance. *Genetics* 2002;162(4):1885-1895.

Kang, Y.*, et al.* Genome‐wide association of drought‐related and biomass traits with HapMap SNPs in Medicago truncatula. *Plant, Cell & Environment* 2015;38(10):1997-2011.

Kim, B. Hierarchical Association Coefficient Algorithm: New Method for Genome-Wide Association Study. *Evolutionary bioinformatics online* 2017;13:1176934317713004.

Kim, B. and Beavis, W.D. Numericware i: Identical by State Matrix Calculator. *Evolutionary bioinformatics online* 2017;13:1176934316688663.

Kim, B., Beavis, W.D. and Leon, J. Numericware N: Numerator Relationship Matrix Calculator. *The Journal of heredity* 2016;107(7):686-690.

Lipka, A.E.*, et al.* GAPIT: genome association and prediction integrated tool. *Bioinformatics* 2012;28(18):2397-2399.

Money, D.*, et al.* LinkImpute: Fast and Accurate Genotype Imputation for Nonmodel Organisms. *G3 (Bethesda, Md.)* 2015;5(11):2383-2390.

Sanchez, D.L.*, et al.* Genome-wide association studies of doubled haploid exotic introgression lines for root system architecture traits in maize (Zea mays L.). *Plant science : an international journal of experimental plant biology* 2018;268:30-38.

Sanderson, C. Armadillo: An open source C++ linear algebra library for fast prototyping and computationally intensive experiments. 2010.

Team, R.C. R: A language and environment for statistical computing. R Foundation for Statistical Computing, Vienna, Austria. 2013. In.: ISBN 3-900051-07-0; 2014.

Turner, S.D. qqman: an R package for visualizing GWAS results using QQ and manhattan plots. *BioRxiv* 2014:005165.

VanRaden, P.M. Efficient methods to compute genomic predictions. *Journal of dairy science* 2008;91(11):4414-4423.

Xie, W.*, et al.* Parent-independent genotyping for constructing an ultrahigh-density linkage map based on population sequencing. *Proceedings of the National Academy of Sciences* 2010;107(23):10578-10583.

Xu, S. Mapping quantitative trait loci by controlling polygenic background effects. *Genetics* 2013;195(4):1209-1222.

Yu, J.*, et al.* A unified mixed-model method for association mapping that accounts for multiple levels of relatedness. *Nature genetics* 2006;38(2):203.

Zhang, W.*, et al.* PEPIS: A pipeline for estimating epistatic effects in quantitative trait locus mapping and genome-wide association studies. *PLoS computational biology* 2016;12(5):e1004925.
